# Pavlovian Olfactory Fear Conditioning: Its Neural Circuity and Importance for Understanding Clinical Fear-Based Disorders

**DOI:** 10.3389/fnmol.2019.00221

**Published:** 2019-09-19

**Authors:** Marziah Hakim, Andrew R. Battle, Arnauld Belmer, Selena E. Bartlett, Luke R. Johnson, Fatemeh Chehrehasa

**Affiliations:** ^1^School of Biomedical Science, Queensland University of Technology, Brisbane, QLD, Australia; ^2^Institute of Health and Biomedical Innovation, Queensland University of Technology, Kelvin Grove, QLD, Australia; ^3^Mater Medical Research Institute and Queensland Health, Queensland University of Technology, The University of Queensland, Woolloongabba, QLD, Australia; ^4^The University of Queensland Diamantina Institute, Translational Research Institute, Woolloongabba, QLD, Australia; ^5^School of Clinical Sciences, Queensland University of Technology, Brisbane, QLD, Australia; ^6^Division of Psychology, School of Medicine, University of Tasmania, Launceston, TAS, Australia; ^7^Center for the Study of Traumatic Stress, School of Medicine, College of Health and Medicine, Uniformed Services University, Bethesda, MD, United States; ^8^Clem Jones Centre for Neurobiology and Stem Cell Research, Griffith Institute for Drug Discovery, Griffith University, Nathan, QLD, Australia

**Keywords:** memory, olfactory, fear conditioning, neurogenesis, amygdala, neuronal circuits, plasticity

## Abstract

Odors have proven to be the most resilient trigger for memories of high emotional saliency. Fear associated olfactory memories pose a detrimental threat of potentially transforming into severe mental illness such as fear and anxiety-related disorders. Many studies have deliberated on auditory, visual and general contextual fear memory (CFC) processes; however, fewer studies have investigated mechanisms of olfactory fear memory. Evidence strongly suggests that the neuroanatomical representation of olfactory fear memory differs from that of auditory and visual fear memory. The aim of this review article is to revisit the literature regarding the understanding of the neurobiological process of fear conditioning and to illustrate the circuitry of olfactory fear memory.

## Introduction

Odor-evoked memories possess the ability to take an individual back to a significant emotional event (Herz, 1998; Schettino and Otto, 2001; Wilson, 2003; Sevelinges et al., 2007; Nigri et al., 2013). They are an integral component of significant autobiographical memories that can allow us to revisit an experience or an event. This was demonstrated by Proust (1976), a French novelist who took a bite of madeleine biscuit that had been dipped in Linden tea and was flooded with memories of a long-forgotten moment from his childhood. This is now commonly known as the “Proust phenomenon” (Proust, 1976). This association between odor and memory seems to be more potent when the experience holds a higher emotional value (Herz and Cupchik, 1995; Herz, 1997b). Olfaction is known as the sense with the highest association with emotional context, and therefore holds a strong ability to trigger memories that carry high emotional saliency (Herz, 1998; Schettino and Otto, 2001; Wilson, 2003; Sevelinges et al., 2007; Nigri et al., 2013). This is a characteristic feature of odor evoked memories, in comparison to recollections triggered by other sensory stimuli such as visual, auditory and tactile (Abraham et al., 1993; Chu and Downes, 2000; Willander and Larsson, 2006; Miles and Berntsen, 2011).

Experimental evidence suggests that the olfactory components of autobiographical memories are more resilient than the visual and auditory components of the same experience (Engen and Ross, 1973; Herz and Cupchik, 1995; Chu and Downes, 2000; Toffolo et al., 2012). Olfactory stimuli are able to persist despite the degradation of other sensory memory cues (Herz and Cupchik, 1995). Willander and Larsson (2006) found that autobiographical memories triggered by olfactory stimuli tend to be older than memories evoked by other sensory cues. This quality of endurance is what makes odors more effective as retrieval cues for emotional memories than other senses (Willander and Larsson, 2006).

Along with evoking highly emotional memories, it has been found that odors have an unusual effect on contextual memory cues. Many studies have found that odors are superior to other sensory stimuli in facilitating context-dependent memory (Cann and Ross, 1989; Schab, 1990; Smith et al., 1992; Herz, 1998) and the presence of odors at the time of the experience and at the time of recall can significantly enhance the retrieval of learned items (Herz, 1998; Otto et al., 2000). Additionally, there is increasing evidence suggesting that successful contextual conditioning is dependent on changes in the internal state of the subject occurring in the context, such as introducing a change in emotional state (Herz, 1997a; Otto and Giardino, 2001; Muñoz-Abellán et al., 2009). This explains the strength of olfactory contextual cues and its tight association with emotional neural substrates in fear conditioning (Herz, 1997b).

The ability of odors to increase a memory’s evocative strength often poses a threat, as they include “fear” memories (Toffolo et al., 2012). There are a range of anxiety and trauma-related disorders which have an olfactory component (Cortese et al., 2015). Although olfaction has great influence on context associated fear and anxiety-related memory pathologies, the encoding and processing of aversive olfactory conditioning has been investigated limitedly (Otto et al., 2000; Schettino and Otto, 2001; Sevelinges et al., 2004; Valley et al., 2009; Ross and Fletcher, 2018).

Much of the research surrounding fear conditioning and its neuroanatomical changes has been predominantly with auditory and visual stimuli (LeDoux et al., 1986; Campeau and Davis, 1995; Valley et al., 2009; Johnson et al., 2012; Bergstrom et al., 2013; Bergstrom and Johnson, 2014; Daldrup et al., 2015), however odors are especially effective at triggering memories of high emotional saliency and intensity, much more so than other sensory cues (Herz, 1998). Furthermore, the olfactory fear memory pathway differs considerably from auditory and visual pathways (Campeau and Davis, 1995; Bergstrom and Johnson, 2014; Parma et al., 2015).

Therefore, studying the mechanisms of olfactory memory can not only provide insight into normal emotions but also mental disorders, in particular, those relating to fear. The focus of this review is to revisit the olfactory system, explore the literature on the neural circuitry of olfactory fear conditioning (OFC) and the effects of OFC on olfactory neurogenesis.

## Olfactory System

**Figure 1:**
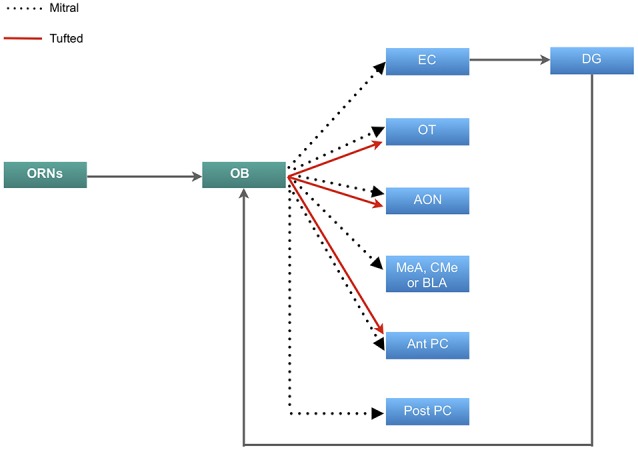
Neural circuitry of olfactory information. Primary olfactory receptor neurons (ORNs) residing in the main olfactory epithelium of the nasal cavity carry information to the olfactory bulb (OB), which is then relayed by the mitral cells to the entorhinal cortex (EC) that sends inputs to the dentate gyrus (DG) and projects back to the OB. Mitral cells also project to the olfactory tubercle (OT), anterior olfactory nucleus (AON), either the medial (MeA), corticomedial (CMe) or basolateral amygdala (BLA) and both the anterior and posterior regions of the piriform cortex (PC). The tufted cells carry olfactory information to the OT, AON and exclusively to the posterior portion of the PC.

The olfactory sense is thought to be the most advanced sensory informant, and by virtue has evolved to a complex system (Eisthen, 1997; Pifferi et al., 2010). The organization of the olfactory system initiates in the olfactory epithelium, which houses primary olfactory receptor neurons (ORNs) and supporting cells. The primary ORNs are bipolar neurons that serve to transmit olfactory information to the olfactory bulb (OB). The unmyelinated axons of these neurons project to the lamina propria where they form large axon bundles which pass through the cribriform plate of the ethmoid bone to arborize and synapse with olfactory second-order neurons on glomeruli within the OB. These second-order neurons are known as mitral and tufted neurons which approximately 20–50 of these neurons emanate from each glomerulus. The axons of mitral and tufted neurons form the lateral olfactory tract which project and synapse at various areas of the olfactory cortex, including the olfactory tubercle (OT), piriform cortex (PC), amygdala, the anterior olfactory nucleus (AON) and entorhinal cortex (EC) which projects to the dentate gyrus (DG) of the hippocampus (Figure 1; Buck, 1996; Lledo et al., 2005; Mori et al., 2006; Illig and Wilson, 2009). The second-order neurons also have lateral dendrites that extend in the glomerular and granular layers of the OB and synapse with olfactory inhibitory interneurons known as periglomerular and granular neurons (Lledo et al., 2008; Bonzano et al., 2016).

The olfactory afferents all function to process, combine and store odor information. The role of the OT is manifold. Odor information reaches the OT predominantly *via* the tufted cells (Scott et al., 1980). The information is modulated by the PC, which receives input predominantly by the mitral cells (Figure 1) (Wesson and Wilson, 2011). This dual input then allows for distinct odor processing. The OT may also play a role in determining the source of an odor and odor discrimination (Wesson and Wilson, 2011). The AON further contributes to odor processing by detecting and storing correlations between olfactory features and creates representations of particular odors and combination of odors. The PC then detects and learns the representations and creates emotional associations (Haberly, 2001). This information is linked to memory *via* the EC. The EC has been found to be responsible for the relational organization of memory and provide differential inputs to the CA1 and CA3 regions of the hippocampus, where memory is consolidated (Figure 1; Eichenbaum et al., 2007; De La Rosa-Prieto et al., 2015; Chaaya et al., 2018). Furthermore, the EC sends olfactory information to the DG of the hippocampus. This connection is reciprocal in that information is carried back to the OB from the ventral subiculum and CA1 (Figure 1; de la Rosa-Prieto et al., 2009; Mohedano-Moriano et al., 2012). Based on these findings, each afferent connection of the OB plays a unique role in olfactory memory processing.

Many studies have explored the trajectory of the mitral and tufted cells from the OB to the PC (Figure 1; Stevens, 1969; Haberly, 1973; Devor, 1976; Schettino and Otto, 2001), which is the largest cortical recipient of direct OB and plays an important role in integrating cognition and experience into odor information stored in the AON (Woolsey and Van der Loos, 1970; Henkin et al., 1977; West and Doty, 1995; Stettler and Axel, 2009). This part of the olfactory cortex has two main subdivisions: anterior PC (APC), and posterior PC (PPC). The ventral part of APC may receive exclusive input from tufted cells (Illig and Wilson, 2009) which discriminates and categorizes the information (Figure 1; Sharp et al., 1977; Stettler and Axel, 2009). Rennaker et al., 2007 showed that odor representations are transformed into ensemble patterns and distributed spatially and temporally in the APC (Rennaker et al., 2007). It was previously believed that the APC was exclusively involved in sensory processes and perceptual learning (Wilson, 1998, 2000, 2003; Rennaker et al., 2007), however more recently, it has been shown that the APC demonstrated post-fear conditional changes, which alludes to its involvement in odor memory recall, along with the PPC (Shepherd, 2010; Barnes et al., 2011; Chen et al., 2011). Furthermore, high resolution functional imaging has shown that the PPC is responsible for coding odor perceptions, a feature that is absent in the APC and amygdala (Howard et al., 2009).

The hippocampus has been studied extensively in regards to its role in spatial and contextual fear memory (CFC; Maren et al., 1998; Wiltgen et al., 2006; Maren, 2014; Zelikowsky et al., 2014; Hersman et al., 2017). Odors form a strong component of context-dependent memory (Herz, 1997a). Several studies have observed significant neuroplastic changes following CFC in the hippocampus (Fanselow, 2000; Han et al., 2016; Schmidt et al., 2017; Chaaya et al., 2018). Interestingly, there have been strong parallels observed between the function of the hippocampus in CFC and the PC in OFC. The hippocampus and PC have comparable circuit organizations in which neuron ensembles that are activated by learning, are necessary for olfactory and contextual memory retrieval (Haberly, 2001; Liu et al., 2012; Meissner-Bernard et al., 2019). Mandairon and colleagues demonstrated that following contextual conditioning, visual context alone was able to activate neural structures similar to that stimulated by odor alone; namely, the PC and OB (Mainland et al., 2014). These findings suggest that independent odor cues and odorants as part of context have similar neural representations. Nevertheless, further research is required to address whether odor-cued fear memories are relayed in the hippocampus directly or as part of contextual memory. This information can provide further insight into whether the circuitry in the PC and hippocampus associated with fear learning are distinct or both are simultaneously activated by olfactory cues.

## Animal Models of Fear Memory

Neuroanatomical studies in animal models and imaging of human brains have showed overlapping results regarding the neurobiology of emotional memories and conditioned fear memories (Delgado et al., 2006; Knapska et al., 2012; Vanelzakker et al., 2014). Therefore, most of the current literature utilizes animal models in order to replicate trauma-related emotional disorders (Wang et al., 2007; Chen et al., 2011; Moore et al., 2012; Russo and Parsons, 2017). One approach to induce fear memory is to introduce physical stressors such as forced swim paradigms, restraint stress, ether exposure and inescapable foot shocks (Vyas et al., 2002; McGuire et al., 2010; Moore et al., 2012; Bergstrom and Johnson, 2014).

Another approach involves psychosocial stressors such as housing instability, social defeat and social isolation. Housing instability is mimicked by changing home cages and cage mates daily, which has been shown to produce long-lasting anxiety-driven behaviors (Zoladz et al., 2012; Saavedra-Rodríguez and Feig, 2013). Social defeat is induced by placing an intruder animal in the territory of a larger resident animal, resulting in the resident attacking the intruder, which produces signs of anxiety in these animals (Huhman, 2006).

The prototypical form of fear learning is Pavlovian fear conditioning (Pavlov, 1927; Maren and Fanselow, 1996). It involves an aversive unconditioned stimulus (US) that evokes an innate negative response, irrespective of training, and a neutral conditioned stimulus (CS). Conditioning involves contingent associations of the CS with the US, such that the CS evokes an aversive conditioned response and predicts the occurrence of the US (Bolles and Collier, 1976). The most common protocol is that an animal receives mild foot shocks with a neutral sensory stimulus (odor, tone or light) concurrently (Johnson et al., 2012). The animal’s learning is then tested by presenting the CS alone, which evokes a behavioral or physiological fear response including increased heart rate and blood pressure and more frequent defecation or urination (Davis, 1992; LeDoux, 1995; Goswami et al., 2013). Another method of measurement is the observation of a fear-potentiated startle response, a reflex that occurs in response to an abrupt acoustic stimulus and results in the rapid contraction of the facial and skeletal muscles (Davis, 1992; Russo and Parsons, 2017). Measuring the duration of freezing (complete immobility except for movements involved in respiration) has also been a common method used in behavioral studies (Bolles and Collier, 1976; Amorapanth et al., 1999; Lee et al., 2005; Daldrup et al., 2015). These are typical unconditioned stress/anxiety responses for rodents, which have evolved into a cogent way to study the development and maintenance of fear and anxiety-related disorders, as they can often follow a similar neurobiological process to Pavlovian fear (Johnson et al., 2012).

## Olfactory Fear Conditioning and Amygdala

In contrast to auditory and visual fear conditioning, fewer studies have investigated olfactory fear conditioning (OFC). Therefore, the neural circuitry of OFC is not fully understood. Some recent studies have begun to focus on the neuroanatomical changes occurring as a result of olfactory fear processing. A study using olfactory fear conditioning using M71 odorant receptor transgenic mice showed that there was an increase in the number of M71 odorant receptors in the nasal cavity and cross sectional of M71 glomeruli of the OB in response to olfactory fear. Furthermore, olfactory extinction training specific to the conditioned odor was shown to reverse the structural changes that occurred as a result of olfactory fear memory acquisition in the olfactory epithelium and the OB (Morrison et al., 2015).

Odorant receptors that are activated by specific odors can be structurally altered by both appetitive and aversive experiences (Ressler et al., 1994; Mori et al., 2006; Fletcher, 2012). Studies have shown that the number of olfactory sensory neurons expressing a specific odorant receptor doubled at 3 weeks post-conditioning and the odor-evoked synaptic outputs of the associated glomeruli were significantly enhanced following associative learning of fear (Kass et al., 2013; Morrison et al., 2015). These studies contribute to the premise that the primary olfactory sensory neurons play a significant role in the sensitivity and responsiveness to a learned cue or CS.

**Figure 2:**
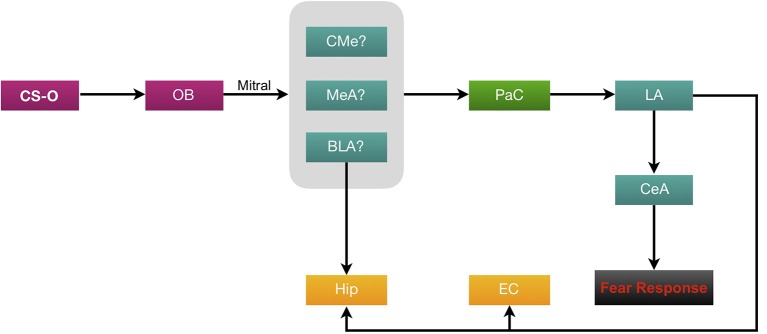
Neural circuitry of olfactory fear conditioning (OFC). Olfactory conditioned stimuli (CS-O) travel through the ORNs to the OB then via the mitral cell axons (forming the olfactory tract), information is carried to the amygdala. Evidence suggests that the likely regions of termination in the amygdala are either the MeA, CMe or BLA. From here, the proposed pathway to the lateral amygdala (LA) is through the periamygdaloid complex (PaC). All CS information is then processed through the central (CeA) amygdala to elicit a fear response. The BLA and LA project heavily to the CA1 and CA3 regions of the hippocampus (Hip). The LA also sends afferents to the EC.

Investigations of the connections between the olfactory system and the amygdala began early in the 20th century (Herrick, 1921). The association of strong emotions with odors is found to be due to olfactory neurons projecting directly to the amygdala; the brain region where memories are given emotional context. Herrick (1921) was one of the first to discover that the amygdala has direct synapses with the OB. Unmyelinated axons of the dorsal olfactory tract are present in the lateral part of the amygdala (Herrick, 1921). Unlike auditory and visual fear conditioning, the olfactory conditioning pathway does not project directly and exclusively to the basolateral amygdala (BLA; Sevelinges et al., 2004; Keshavarzi et al., 2015; Luchkina and Bolshakov, 2018). Schettino and Otto (2001) also investigated the activation of amygdala subregions during the acquisition and expression of OFC. Their results demonstrated that the medial (MeA) and corticomedial (CMe) nuclei of the amygdala are activated by odor and shock, both individually and paired. Further refining its role (Walker et al., 2005) infused the NMDA receptor antagonist AP5 and the α-amino-3-hydroxy-5-methyl-4-isoxazolepropionic acid (AMPA)/kainate receptor antagonist 2,3-dihydroxy-6-nitro-7-sulfamoyl-benzoquinoxaline (NBQX) into the MeA, which ceased fear-potentiated responses to olfactory CS. This indicated the involvement of the MeA in eliciting fear expression behaviorally as opposed to being involved in fear memory consolidation. It is believed that fear conditioning induces structural changes in the lateral nuclei of the amygdala (LA), underlying acquisition and storage of CS and US or shock associations (LeDoux, 1995). Since the LA plays an integral role in the convergence of CS and US information, it is crucial for olfactory information to reach this region of the amygdala (Romanski et al., 1997; Johnson et al., 2012). Schettino and Otto (2001) proposed that the most likely pathway to the LA is through the periamygdaloid complex (PaC; Figure 2).

The role of the central amygdala (CeA) has been studied well in a review article by Keifer et al. (2015). This nucleus is subdivided into the centromedial (CEm) and centrolateral (CEl) nuclei. It has been suggested that the CEl plays a role in fear learning, whereas the CEm is more involved in fear output (Ciocchi et al., 2010). The CeA has inhibitory connections with the prefrontal cortex (PFC), a region which is activated in response to recall of an extinguished fear (Quirk et al., 2003; Milad et al., 2007). However, this has not yet been studied with OFC. While the exact role of the subnuclei of CeA in OFC is not fully understood, lesioning of CeA was shown to abolish OFC (Hitchcock et al., 1989; Sananes and Campbell, 1989). Additionally, a single study has shown that OFC also induces field potential signaling in the cortical amygdala (CoA; Sevelinges et al., 2004).

Furthermore, the BLA has also been responsive to OFC. *In vivo* intracellular recordings in the BLA have demonstrated that repeated odor-shock pairings resulted in enhanced post-synaptic potential responses to the odor (Rosenkranz and Grace, 2002). Similarly, Kilpatrick and Cahill (2003) found that inactivation of the BLA following olfactory conditioning resulted in a learning deficit, suggesting the involvement of the BLA in odor fear memory consolidation. These results were similar to (Cousens and Otto, 1998), who found that pre-training, excitotoxic lesions of the BLA abolished conditioned freezing to an odor. Overall, results from studies regarding the involvement of subregions of the amygdala in OFC have been highly multifarious in comparison to auditory and visual fear conditionings. Further study on OFC and investigation of the specific roles of the subregions of the amygdala could give significant insight on this field.

The amygdala has several connections to the EC and hippocampus, which further contributes to its connotation of memory consolidation. An investigation of its projections to the hippocampal formation revealed that the LA primarily projected to the ventral and dorsal intermediate entorhinal subfield (VIE and DIE, respectively; Pikkarainen et al., 1999). Part of the VIE and DIE also had light projections originating in the basal nucleus of the amygdala (BA). In addition, the BA and LA projected, respectively, to the CA1 and CA3 regions of the ventral hippocampus (Pikkarainen et al., 1999; Chaaya et al., 2018). These projections carry information of CFC to the EC and hippocampus where it undergoes consolidation (Maren and Fanselow, 1997; Chaaya et al., 2018). Further research investigating these projections from the amygdala to the hippocampal formation in relation to OFC would clarify its role further. Due to overlapping neuroanatomy thus found in OFC and CFC, it would be innocuous to the hypothesis that the connections of the amygdala would also overlap.

## Neurogenesis in Response to Olfactory Fear Learning

A striking aspect of the olfactory system is that it constantly receives supply of newborn interneurons from the (SVZ) directly to the OB (Sultan et al., 2010). Additionally, the ORNs lining the nasal cavity are capable of regenerating throughout life (Mackay-Sim and Kittel, 1991; Chehrehasa et al., 2005, 2010). Being under constant environmental detriment, ORNs have a short lifespan of 30–90 days (Mackay-Sim and Kittel, 1991). There are two cell populations in the basal layer of the olfactory epithelium, the horizontal basal cells and the globose basal cells. These cells are responsible for regeneration in the olfactory epithelium (Iwai et al., 2008). While regeneration of primary olfactory neurons is well understood, it is unclear how they respond to OFC and whether the regeneration of the primary olfactory neurons is affected by olfactory fear memory.

Introduction of thymidine analogs such as bromodeoxyuridine (BrdU), which labels newborn cells, contributed significantly to the field of adult neurogenesis (Biffo et al., 1992; Kempermann et al., 2003; Sadgrove et al., 2006; Kondziella et al., 2011). More recently, 5-ethynyl-2′-deoxyuridine (EdU), a new thymidine analog, was tested *in vivo*, which confirmed neurogenesis of the brain and of the olfactory system (Salic and Mitchison, 2008; Chehrehasa et al., 2009). Results of DNA labeling indicated that newborn neurons from the SVZ of the lateral ventricles continually migrated through the rostral migratory stream (RMS) to the OB (Gheusi and Lledo, 2014). As a result, the newborn interneurons continually integrate into the OB circuit (Sakamoto et al., 2014). In rodents, when neurogenesis of the OB was inhibited by various methods, such as a bulbectomy (removing the OB), SVZ ablation or suppression of neuroblast proliferation, olfactory memory retention was significantly impaired and learning of olfactory cues was delayed (Jaako-Movits and Zharkovsky, 2005; Valley et al., 2009). However, the significance of the olfactory inhibitory interneurons and postnatal neurogenesis on aversive olfactory learning and behavior remains inconclusive.

Interestingly, a study has shown that a bilateral bulbectomy decreased granule cell proliferation in the DG of the hippocampus, demonstrating the interconnectedness of the two structures (Jaako-Movits and Zharkovsky, 2005). Since the hippocampus is known to act as an input structure and processing house for visuospatial, auditory and olfactory information, it has been proposed that the OB affects the acquisition of context and formation of stable connections between olfactory components of contextual cues and US (Jaako-Movits and Zharkovsky, 2005).

Contrastingly, a study conducted by So et al. (2008) showed that OFC increased proliferation of neuroblasts in the SVZ, but not the DG. The study controlled for intrinsic contextual learning and therefore the results were only an effect of odor cued conditioning. Furthermore, there is a clear link between neurogenesis in the DG and contextual fear conditioning, however not OFC (Saxe et al., 2006). Despite the structural association between the OB and DG (Figure 1), olfactory and contextual learning tasks have shown to independently induce proliferation in either the SVZ or the DG, respectively. Together, these studies show that there is a distinction between the neurogenesis involved in contextual fear conditioning and in cued OFC. However, there is still a need for further research to gain strong evidence on the effect on neurogenesis in the DG following OFC.

Increasing adult hippocampal neurogenesis has been shown to improve pattern separation. Sahay et al. (2011) selectively increased adult neurogenesis in the hippocampus and found that the mice were able to better distinguish between two similar contexts. Similarly, neurogenesis occurring in the OB functions to facilitate the distinction between odors. The survival and integration of newborn neurons enhance odor acuity following olfactory conditioning (Rochefort et al., 2002; Moreno et al., 2009). With this evidence, we can conclude that neurogenesis plays an adaptive role to optimally encode contextual and olfactory information.

The role of newborn olfactory granule cells in regard to learning and memory was further explored by analyzing neuronal activity of granule cells through electrophysiological recordings (Nissant et al., 2009). Nissant et al. (2009) investigated synaptic plasticity of newborn and mature granule cells in the OB following theta-burst stimulation which induced long term potentiation (LTP). This study showed that LTP-related plasticity could not be induced in mature granule cells in postnatal mice but was transiently induced in newborn granule cells in adults. This suggests that young adult-born neurons in the OB are particularly susceptible to synaptic plasticity related to LTP and proved that neurogenesis contributes to the retention of long-term memory.

Both perceptual and associative olfactory learnings cause selective recruitment of newborn neurons to the OB (Moreno et al., 2009; Sultan et al., 2010). At approximately 2–4 weeks following thymidine analog labeling, it was found that the newborn neurons underwent an elimination process and approximately half of the newly-generated cells encountered apoptosis (Rochefort et al., 2002; Dayer et al., 2003; Kempermann et al., 2003). The selection of the surviving neurons has been positively correlated with olfactory activity (So et al., 2008; Valley et al., 2009; Sultan et al., 2010). Reconstruction of spatial density maps of the granule cell layer of the OB demonstrate that recall of an associative task recruits young neurons in odor-specific areas of the OB and their survival is proportional to the strength of learning (Sultan et al., 2010). Although this study shows the importance of olfactory learning on survival of newborn neurons, it utilized a reward conditioning paradigm, which may not accurately represent fear memory and may not apply to anxiety-related pathologies.

Further experiments would be required to clarify the role of brain neurogenesis in olfactory fear learning and memory; such as the mechanisms underlying long term memory compared to that of short-term memory and the incorporation of adult-born neurons into the afferent connections of the OB.

## Conclusion

The aim of this review article was to revisit the literature on OFC in order to understand the neuronal circuitry of olfactory fear memories. Recent studies showed that the amygdala, PC and OB are essential regions involved in olfactory fear memory processing. In addition, a small body of literature also suggests that the amygdala subnuclei, MeA, CMe, CeA and BLA, have a degree of involvement in the consolidation and expression of olfactory fear memory, however, this is not fully understood due to limited research in the area. Further experimental studies are required to determine which subnuclei of the amygdala and which subregions of the PC are involved in OFC and to understand their specific role in olfactory fear memory processing. In addition, it has been shown that olfactory fear conditioning results in an increase in neurogenesis in the SVZ. Moreover, newborn neurons are potential key players in the retention of long term memory, however, the specific role of the newborn neurons is yet to be investigated.

Overall, in order to provide efficient therapeutic strategies for patients with fear-related mental disorders, it is essential to understand the underlying neuronal circuits. Importantly, it has been shown that exposure therapy for fear-related disorders has not had long term efficacy due to the influence of context, which includes odor (Bouton, 2004). From this knowledge, a holistic and more targeted approach for treatment of fear memory-related disorders is needed, that includes all sensory inputs.

## Author Contributions

FC, ARB and LJ devised the study context. MH and FC conducted the literature review, selected the articles, and drafted the manuscript. FC, ARB, LJ, SB, and AB revised the manuscript. All authors revised the manuscript for intellectual content.

## Conflict of Interest

The authors declare that the research was conducted in the absence of any commercial or financial relationships that could be construed as a potential conflict of interest.
